# Treatment of large chondral lesions with an autologous minced cartilage technique and synovial flap leads to superior results compared to matrix associated autologous chondrocyte transplantation technique after 24 months: A controlled clinical trial

**DOI:** 10.1002/ksa.12708

**Published:** 2025-05-26

**Authors:** Johanna Mayr, Franziska Warth, Nicola Oehler, Martin Majewski, Christoph Lutter, Fabian Blanke

**Affiliations:** ^1^ Department of Knee‐, Hip‐, Shoulder‐, Elbow Surgery and Orthopedic Sports Medicine, Schön Klinik München Harlaching FIFA Medical Center Munich Germany; ^2^ Department of Orthopedic Surgery University of Rostock Rostock Germany; ^3^ Department of Orthopedic Sports Medicine and Arthroscopic Surgery Hessing Stiftung Augsburg Augsburg Germany; ^4^ Department of Orthopedic Surgery University of Basel Basel Switzerland

**Keywords:** autologous, knee joint, large lesions, minced cartilage, synovial flap

## Abstract

**Purpose:**

Treating large cartilage lesions in the knee remains a challenge. While matrix‐associated autologous chondrocyte implantation (MACI) is the gold standard for medium to large lesions, the minced cartilage technique has shown promise in smaller defects. Enhancing this technique with biomaterials has been suggested for larger lesions, but its effectiveness remains unclear due to limited data. This study aimed to evaluate the outcomes of the minced cartilage technique with autologous synovial flap coverage in large knee cartilage lesions and compare the results with MACI.

**Methods:**

Twenty patients with large Grade III–IV cartilage defects (>6 cm²) at the knee were included. Ten patients underwent the autologous minced cartilage procedure (AutoCart™) with synovial flap (Group A), and ten received the MACI procedure (Group B). Clinical outcomes were assessed using the Tegner score, visual analog scale (VAS), the International Knee Documentation Committee (IKDC) forms, and the Knee Injury and Osteoarthritis Outcome Score (KOOS). MRI evaluations were performed using the MOCART 2.0 score before surgery and 24 months postoperatively.

**Results:**

Clinical scores significantly improved in Group A after surgery, while Group B showed improvement only in the VAS, pain, and sports/recreation levels. Postoperative MRI revealed similar results between groups, with Group A showing significantly better cartilage defect volume fill and fewer subchondral changes compared to Group B (*p* < 0.05). The mean MOCART 2.0 score at the final follow‐up was 76.0 ± 15.4 for Group A and 65.6 ± 17.6 for Group B, though without statistical significance.

**Conclusion:**

The study suggests that the all‐autologous minced cartilage technique with synovial flap is an effective treatment for large chondral lesions, yielding outcomes similar to or better compared to the MACI technique.

**Level of Evidence:**

Level III.

**Abbreviations**
ACL: anterior cruciate ligament
ADL: activities of daily living
ICRS: international cartilage regeneration & joint preservation society
IKDC: international knee documentation committee
KOOS: knee injury and osteoarthritis outcome score
MACI: matrix‐associated autologous chondrocyte implantation
MOCART: magnetic resonance observation of cartilage repair tissue
MRI: magnetic resonance imaging
PACS: picture archiving and communication system
PDw‐TSE: proton‐density‐weighted–turbo spin‐echo
PRP: platelet rich plasma
QoL: quality of life
SD: standard deviation
VAS: visual analog scale

## INTRODUCTION

The treatment of large cartilage lesions is still a challenge for orthopedic knee surgeons. Currently, different surgical treatment options are used, mostly depending on the surgeon's preference [13, 25]. Microfracturing is a satisfying technique for small lesions but has its limitations in medium and large lesions [27, 28]. Therefore, matrix‐associated autologous chondrocyte implantation (MACI) has evolved to become the gold standard procedure for medium and large lesions due to superior tissue quality and preservation of the subchondral bone [7, 25, 27]. Nonetheless, the two‐step procedure and the high perioperative costs of MACI are significant disadvantages [15, 24]. The minced cartilage procedure has gained more attention in recent years for the treatment of cartilage lesions [5, 30, 33]. Promising results have been obtained in small and medium lesions, and many surgeons have recognised the benefits of this whole autologous one‐step procedure [3, 9, 10, 20, 21, 22, 30, 34]. However, it remains unclear whether this technique is effective in large lesions due to the uncertain viability of minced chondral cells and the quality of repair tissue achieved [5, 9, 30]. The application of stem cells or biomaterials to the minced transplant is being discussed to improve cell regeneration in larger lesions but the data is controversial [1, 2, 16, 20, 34]. However, covering the minced transplant with a synovial flap in larger lesions might be a very promising technique because of the availability of this autologous tissue and the good tissue enhancing effects in animal studies due to the high differentiation potential of the synovium [11, 17, 35]. Therefore, the goal of this retrospective study was to evaluate the outcome of an all autologous minced cartilage technique with synovial flaps in large cartilage lesions and compare the results with the MACI technique in same lesions at the knee joint. To our best knowledge data about this topic is lacking in recent literature. We hypothesised that the minced cartilage technique with synovial flap leads to similar clinical and radiological outcome results in large cartilage lesions compared to the MACI technique.

## METHODS

### Study design and study group

A retrospective controlled cohort study was performed. All patients gave their informed consent and institutional review board approval was obtained. Inclusion criteria were patient age >18 years, an isolated focal cartilage defect Grade IV ≥ 6 cm^2^ diagnosed by magnetic resonance imaging (MRI) and arthroscopically. Subsequent surgical treatment by minced cartilage technique and synovial flap coverage or matrix‐associated autologous chondrocyte transplantation (MACT) were performed. Patients with radiologically apparent degenerative joint disease, bifocal cartilage lesions > Grade II to ICRS or meniscal lesions, malalignment in the knee (>3° varus or valgus), instability of the collateral/cruciate ligaments, total/subtotal resected meniscus, vascular disorders (peripheral arterial disease), or inflammatory arthritis were excluded from the study. The following concomitant surgeries were performed: plica resection *n* = 4. In all patients, minced cartilage procedure or MACT was the first‐line treatment. Preoperative diagnostics included clinical examination and MRI of the affected knee. Clinical assessments were performed by two experienced orthopedic surgeons (F.B. and N.O).

### Surgical techniques and rehabilitation

Surgical interventions were performed by one experienced surgeon (F.B).

#### Minced cartilage technique with synovial flap

**Figure 1:**
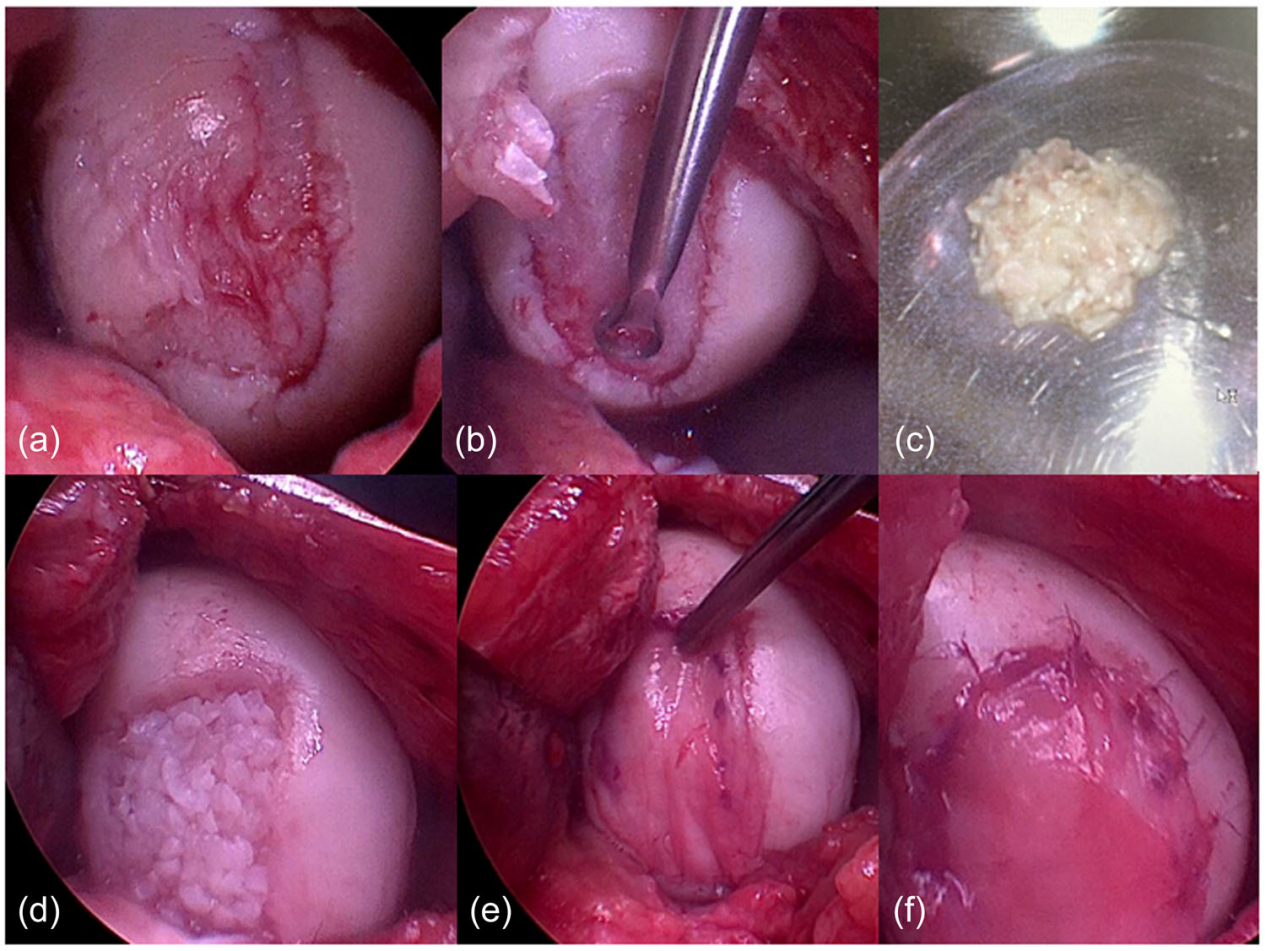
Steps of minced cartilage technique with coverage by a synovial flap. After debridement of the large defect (a, b) and mincing of cartilage tissue from the healthy rim (c), the minced cells are transplanted in the chondral defect (d) and covered with an autologous synovial flap (e). The flap is sutured to the rim of the defect with 6.0 PDS (f).

The technique described here was applied at the patella, trochlea, and both femoral condyles. The presented surgical description is for the femoral condyle, and identical for all locations (Figure 1). The patient position was supine. A tourniquet was used to implant the cartilage under bloodless settings. Since autologous platelet‐rich plasma (PRP) will be required for the procedure, it is recommended to draw venous blood from the patient (e.g., cubital veins) under absolutely sterile conditions before initiation of anaesthesia or at side without cannula placement (to avoid detrimental effects of narcotic substances on the PRP). It is suggested to collect at least 10–15 mL of pure PRP. The PRP was then further processed during arthroscopy. Every indicated minced cartilage procedure was initiated via standard arthroscopy of the index knee joint including possible cointerventions. The intended‐to‐treat cartilage defect was well inspected, and final indication was given during arthroscopy. The size of the defect was measured after debridement. All transplantations were performed in mini‐arthrotomy technique. The cartilage defect was debrided in standardised fashion by using a small sharp spoon/ringed curette. The technique for an optimal debridement of cartilage lesions creating a stable wall and viable rim has been described in detail previously [29]. The calcified layer was gradually debrided and tried to keep intact; however, it remains a matter of debate [32]. Microfracture or microdrilling of the subchondral bone for influx of blood into the defect is not recommended. Contamination of the transplant by the blood clot can negatively affect the chondral fragments and does not provide a significant amount of mesenchymal stem cells [32]. The cartilage graft was harvested from the defective cartilage itself in the cases of acute traumatic chondral lesions when the cartilage clearly appeared healthy and had only lately been delaminated. Degenerative cartilage from the defect or tissue from the intercondylar notch was not utilised for further transplantation. The typical harvesting site in the present study was the healthy rim of the cartilage defect, and the minor enlargement of the defect of approximately 1 mm circumferentially was tolerated. Cartilage was harvested by use of a curette or a 3.5‐shaver device. Beforehand, a collecting device (e.g., GraftNet; Arthrex, GmbH, Munich, Germany) autologous tissue collector was connected to the shaver for harvesting. In most of the cases the cartilage fragments were harvested by a curette, collected in a water‐filled bowl and minced with the 3.5 shaver afterwards. Subsequently, the minced cartilage was mixed with 2–3 drops of PRP outside the patient in a bowl, resulting in a malleable substance. Then, 3 mL and 15–20 min later 6 mL of the PRP was inserted into a specific device (Thrombinator; Arthrex, GmbH) and was gently mixed. In the meantime the prepared defect was inspected. Refinement of defect preparation might be performed with a curette (Figure 1). Then a synovial flap was harvested by the size of the prepared cartilage defect from the medial knee compartment. In the next step, thrombin that was just collected from the Thrombinator was applied drop by drop over the chips‐paste. The chips/PRP/thrombin‐paste was distributed over the defect for complete coverage in an open technique. The consistency of the chips‐paste provided initial stability, because the thrombin combines with the PRP within the chips and generated fibrin, which coagulates quickly and finally fixed the chips within the cartilage defect. The tissue was sealed with a final layer of fibrin that was mixed together previously at the back table. Then minced cartilage transplant was covered by the synovial flap and sutured to the surrounding cartilage rim with resorbable sutures (PDS, 6.0, Fa. Ethicon) (Figure 1). The filling of the treated defects with the cartilage transplant achieved at least 80% of the height of the surrounding healthy cartilage in all cases which made the suturing of the synovial flap more easily (Figure 1). The procedure was then finished. A drain was not applied. The leg was placed into full extension and immobilised in a straight brace. The straight brace was changed to a hinged knee brace after 2–7 days. Partial weight‐bearing of 10 kg was performed for 2–6 weeks due to the location of the lesion.

#### Matrix‐associated chondrocyte transplantation

A two‐stage approach was conducted for each patient. During the initial arthroscopic surgery of the affected knee joint, the cartilage defect was investigated and measured, and indication for MACT was reconfirmed with minimum lesion size of 2 cm^2^. A cartilage biopsy specimen was harvested with a punch (4‐mm diameter) from the noneweight‐bearing femoral trochlear/intercondylar notch of the knee. The specimen was then sent together with an autologous blood sample of 9 mL to the manufacturer (TETEC Tissue Engineering Technologies AG). The second all arthroscopic procedure was performed after a mean time period of 28 ± 4.8 days (range 22–42). Knee position of the patient varied depending on the location of the chondral defect. Lesions of the trochlea were transplanted with an extended knee, whereas femoral lesions were treated in 90°–120° flexion. The lesion was debrided to produce stable perpendicular margins without affecting the subchondral bone. After stopping the fluid irrigation to keep the defect dry, the chondral defect was carefully filled with Novocart inject, a combination of two components, A and B, in an arthroscopically or mini open technique. Component A consisted of the autologous cartilage cells suspended in a solution containing modified human albumin (maleimido‐albumin, human serum albumin [MAHSA]), isotonic sodium hyaluronate, human serum, and cell culture media. Component B contained a cross‐linking component consisting of a,u‐bisthio‐polyethylene glycol in solution. Using a special injection system with a dual‐chamber syringe provided by the manufacturer (TETEC Tissue Engineering Technologies AG), these two components were mixed during the application, resulting in a cross‐linked hydrogel at the site of administration. The application was performed until the cartilage defect was completely filled and the hydrogel fitted flush with the margins of the cartilage margins (2.5 mm approximate application height). Polymerisation was completed after 30–60 s, and the bioresorbable hydrogel bonded immediately to the bottom of the defect and kept the cells at the defect location without the need of further fixation. Polymerised hydrogel contains 0.4–1.6 million chondrocytes per cm^2^.

For both surgical techniques all patients adhered to a standardised postoperative rehabilitation protocol. For patients with cartilage defects located on the femoral condyle or the tibial plateau, this consisted of the noneweight‐bearing of the operated extremity for 6 weeks. After maintaining a stretched position for 48 h, range of motion was restricted from 0° up to a flexion of 60° for 2 weeks postoperatively using a hinged brace (Collamed; Medi GmbH & Co, Bayreuth, Germany). For patients with chondral defects of the trochlea, rehabilitation consisted of a restricted range of motion of 0°–30° flexion within the first 3 weeks and 0°–60° flexion for another 3 weeks after surgery using the same hinged brace. Full weight‐bearing was allowed after 6 weeks. Return to sports was allowed 6 months after surgery.

### Outcome scores

All patients were evaluated preoperatively and at 24‐months follow‐up, including physical examination and completion of a questionnaire. The subjective and functional scores applied to evaluate the clinical outcome included the Tegner Score, visual analog scale (VAS), and the subjective and objective evaluation form of the International Knee Documentation Committee (IKDC). Also, the Knee Injury and Osteoarthritis Outcome Score (KOOS) assessing knee pain, symptoms, activities of daily living (ADL), sports and recreation, and knee‐related quality of life (QoL) was assessed. Clinical relevance was assessed applying minimal clinically important difference (MCID), minimal detectable change, and minimal important change according to recent literature.

### MRI evaluation

MRI of the respective knee joint was conducted before surgery and at 24 months final follow‐up. Since MRI scans were part of the routine clinical follow‐up and not conducted for a prospective study, sequence parameters varied slightly. Yet, the majority of the patients underwent MRI examination on the same 3.0‐Tesla scanner (Avanto; Siemens Medical Systems, Erlangen, Germany) using an 8‐channel phased‐array extremity coil. The following sequences were performed: (1) sagittal fat‐saturated (fs) proton‐density‐weighted turbo spin‐echo (PDw TSE) sequence, (2) a sagittal T1‐weighted TSE, (3) a coronal fs PDw TSE sequence and for patients with trochlear chondral defects, and (4) an axial fs PDw TSE sequence, respectively. The MRI scans were evaluated using the Infinitt PACS viewer (Infinitt Europe GmbH, Frankfurt, Germany). Size of the lesions were determined with the PACS measuring tool. MRI scans were analysed using the MOCART (Magnetic Resonance Observation of Cartilage Repair Tissue) 2.0 Knee Score, a recently published update on the original MOCART score [4]. This revised version integrates seven variables (volume fill of cartilage defect, integration into adjacent cartilage, surface of the repair tissue, structure of the repair tissue, signal intensity of the repair tissue, bony defect or bony overgrowth, subchondral changes). Overall, a score ranging from 0 to a maximum of 100 points may be reached. MRI evaluations were performed by two independent experienced clinicians with musculoskeletal subspecialty. After an initial blinded assessment, all images were reviewed in consensus.

### Statistical analysis

Statistical analysis was performed using GraphPad Prism 7 software (GraphPad software, San Diego, USA). Continuous and categorical variables were expressed as mean ± standard deviation (range) and *n*, respectively. Values of demographics, clinical data/scores and radiological measurements were compared using both paired or unpaired Student's t‐test depending on the respective subgroup analysis. *p*‐Values < 0.05 were considered statistically significant. Normal distribution was tested using D'Agostino and Pearson normality test. Post hoc power analysis using the G*Power 3.1 (HHU Düsseldorf) calculator for paired student's t‐test incorporating an alpha level of 0.05 (95% confidence) demonstrated a statistical power of >0.8 based on calculated effect sizes (cohen's *d*z) for VAS.

## RESULTS

Ten patients with a cartilage defect > 6 cm^2^ and treated by the minced cartilage technique (Group A) and 10 patients with a cartilage defect > 6 cm^2^ and treated by the MACI technique (Group B) were included. Detailed patient characteristics are displayed in Table 1.

**Table 1:** Patient characteristics in Group A and Group B.

Demographic data/baseline characteristics	Minced cartilage + SF	MACI
Patients (*n*)	10	10
Gender (male/female, *n*)	5/5	7/2
Age (years, mean ± SD, range)	41.1 ± 9.4 (28–54)	43.4 ± 5.7 (37–53)
Follow‐up (months ± SD, range)	24.9 ± 1.0 (24–27)	25.3 ± 1.2 (24–27)
Side affected (left/right, *n*)	6/4	2/7
Defect localisation (*n*)		
Medial femur condyle	5	4
Lateral femur condyle	1	1
Medial tibial plateau	0	0
Lateral tibial plateau	0	0
Retropatellar	3	0
Trochlea	1	5

Abbreviations: MACI, matrix‐associated autologous chondrocyte implantation; SD, standard deviation; SF, synovial flap.

All clinical scores showed significant improvement in Group A after surgical intervention.(Table 2). In Group B only VAS score, pain and sport/recreations level improved (Table 3). Apart from pain all clinical scores were significantly superior in group A compared to Group B (*p* < 0.05).

**Table 2:** Clinical scores before treatment and at final follow up in Group A.

	Minced cartilage + SF				
	Baseline		Follow‐up		
Score	Mean ± SD	Range	Mean ± SD	Range	*p* value
VAS	6.0 ± 0.7	5.0–7.0	0.6 ± 0.8	0.0–2.0	<0.0001
Tegner	2.2 ± 0.4	2.0–3.0	5.8 ± 1.5	4.0–7.0	<0.0001
IKDC	37.2 ± 2.2	34.5–40.2	77.0 ± 16.6	52.9–97.7	<0.0001
KOOS					
Pain	50.6 ± 5.2	44.5–58.3	82.3 ± 14.7	56.0–94.4	<0.0001
Symptoms	39.3 ± 5.3	32.2–46.4	72.9 ± 16.1	46.4–89.3	<0.0001
ADL	37.7 ± 6.7	28.0–45.7	80.9 ± 12.1	60.3–92.7	<0.0001
Sports and recreation	9.0 ± 3.9	5.0–15.0	67.0 ± 22.5	30.0–90.0	<0.0001
QoL	11.25 ± 4.9	6.25–18.75	66.3 ± 20.24	37.5–87.5	<0.0001

Abbreviations: ADL, activities of daily living; IKDC, international knee documentation committee; KOOS, knee injury and osteoarthritis outcome score; QoL, quality of life; SD, standard deviation; SF, synovial flap; VAS, visual analog scale.

**Table 3:** Clinical scores before treatment and at final follow up in Group B.

	MACI				
	Baseline		Follow‐up		
Score	Mean ± SD	Range	Mean ± SD	Range	*p* value
VAS	6.1 ± 3.5	1.0–10.0	2.8 ± 1.5	0.5–5.0	0.0081
Tegner	2.9 ± 1.4	1.0–5.0	3.7 ± 1.1	2.0–6.0	ns
IKDC	48.5 ± 23.1	9.2–78.2	55.3 ± 18.5	27.6–78.2	ns
KOOS					
Pain	50.0 ± 22.7	11.0–83.3	69.1 ± 21.3	36.0–94.5	0.0199
Symptoms	46.1 ± 16.5	10.8–60.8	49.2 ± 15.9	28.5–71.5	ns
ADL	64.2 ± 24.6	8.8–95.5	75.6 ± 15.6	44.3–92.3	ns
Sports and recreation	28.9 ± 17.8	0.0–55.0	60.6 ± 18.8	25.0–85.0	0.0005
QoL	40.3 ± 34.8	0.0–100.0	44.4 ± 14.3	12.5–62.5	ns

Abbreviations: ADL, activities of daily living; IKDC, international knee documentation committee; KOOS, knee injury and osteoarthritis outcome score; MACI, matrix‐associated autologous chondrocyte implantation; QoL, quality of life; SD, standard deviation; VAS, visual analog scale.

**Figure 2:**
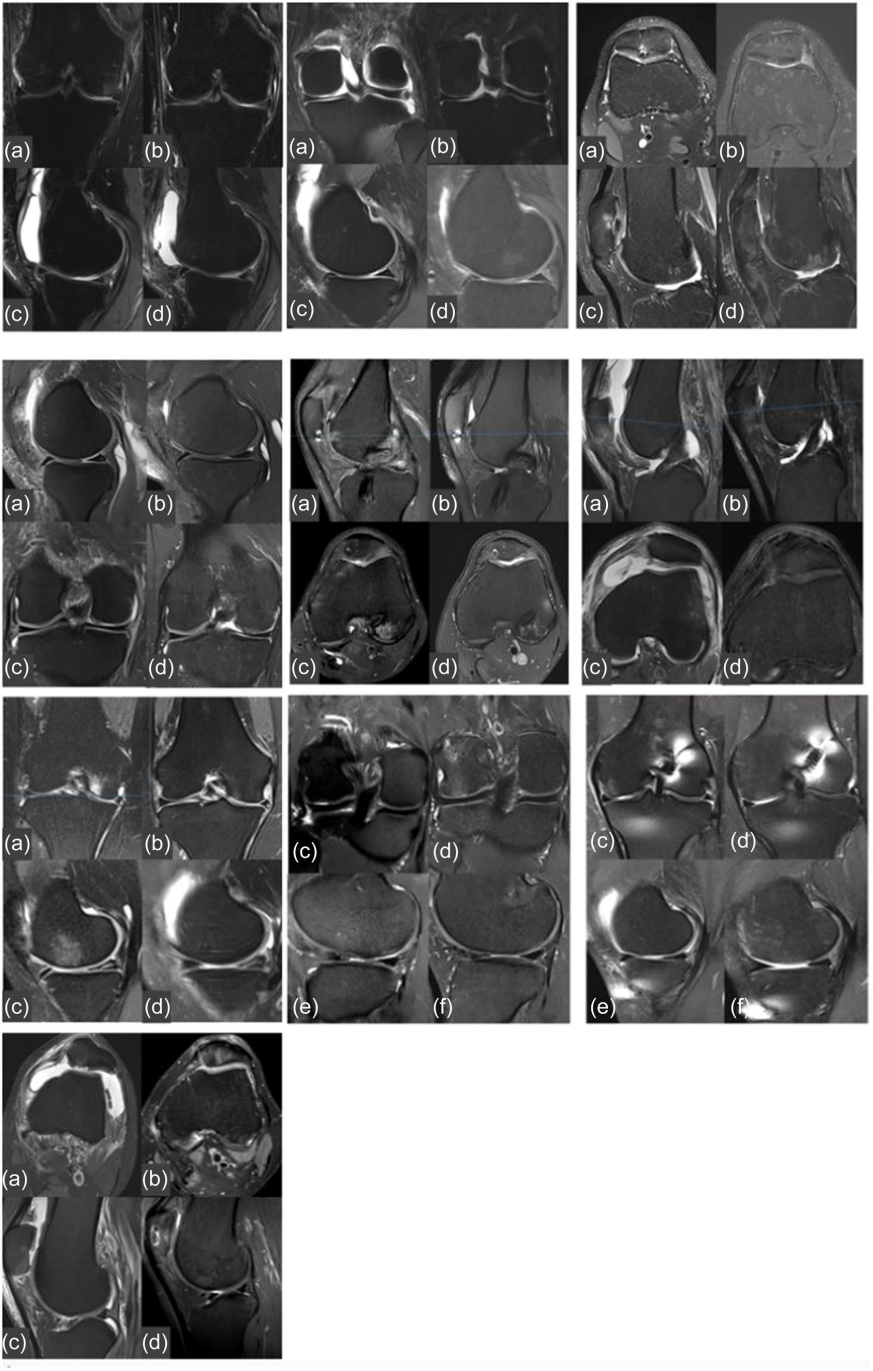
MRI of all patients before surgery (a, c) and 24 months after surgical treatment (b, d). MRI, magnetic resonance imaging.

However, postoperative MRI evaluation showed almost similar results between both groups. Only volume fill of cartilage defect and subchondral changes were significantly better in Group A compared to Group B (*p* < 0.05) (Table 4 and Figure 2).

**Table 4:** Comparison of MRI scores (MOCART 2.0) between Group A and Group B at final follow up.

	Minced cartilage + SF		MACT		
MOCART2.0 score subdomains (points)	Mean ± SD	Range	Mean ± SD	Range	*p* value
Volume fill of cartilage defect (0–20)	18.0 ± 4.2	10.0–20.0	11.7 ± 7.1	0.0–20.0	0.0280
Integration into adjacent cartilage (0–15)	13.0 ± 2.6	10.0–15.0	13.3 ± 2.5	10.0–15.0	ns
Surface of the repair tissue (0–10)	5.0 ± 3.3	0.0–10.0	6.7 ± 2.5	5.0–10.0	ns
Structure of the repair tissue (0–10)	4.0 ± 5.2	0.0–10.0	5.6 ± 5.3	0.0–10.0	ns
Signal intensity of the repair tissue (0–15)	8.0 ± 4.2	0.0–10.0	8.3 ± 5.0	0.0–15.0	ns
Bony defect or bony overgrowth (0–10)	8.0 ± 2.6	5.0–10.0	7.2 ± 2.6	5.0–10.0	ns
Subchondral changes (0–20)	20.0 ± 0.0	20.0–20.0	11.7 ± 9.4	0.0–20.0	0.0116
**Total (0‐100)**	**76.0** ± **15.4**	**50.0–95.0**	**65.6** ± **17.6**	**40.0–85.0**	**ns**

Abbreviations: MACT, matrix‐associated autologous chondrocyte transplantation; MOCART, magnetic resonance observation of cartilage repair tissue; MRI, magnetic resonance imaging; SD, standard deviation; SF, synovial flap.

## DISCUSSION

Present study showed that an all‐autologous minced cartilage procedure with synovial flap is an effective treatment method in large chondral lesions and similar or even superior outcome results can be achieved compared to the MACI technique in these patients.

The minced cartilage procedure is a promising autologous one‐step technique, which showed good clinical and radiological outcome results in small chondral lesion independently from the defect location [3, 9, 10, 12, 22, 31]. This technique can be performed arthroscopically and by arthrotomy without external tissue engineering [30, 32]. Therefore, it is an “in situ tissue engineering technique” with low costs and can be performed spontaneously if a chondral defect is detected. The technique was first described in the early 1980s and modified by different authors [30]. Recent studies showed good clinical results with improvement of clinical scores and satisfying radiological outcome [3, 8, 12, 22, 30, 31]. However, there are some crucial doubts which are linked to the minced cartilage technique. First of all the harvesting of the cells is not always easy and might be contaminated with necrotic or damaged chondral cells [2, 36]. Moreover, it is not clear which quality of tissue can be achieved in the long term follow up. There is evidence that viable chondral cells can be preserved by cutting the cartilage quick and sharply in the smallest possible chips with a scalpel or special shaving blade [6, 31]. The tissue quality confirmed by histological examination was comparable to ACI and superior to microfracturing in animal models [9, 14, 23, 31]. Nonetheless, treatment of large chondral lesions was critically assessed because the data about this procedure is limited and the MACI represents a proven technique for all sizes of chondral lesions in an open or arthroscopic approach and with verified information about the vital cell proportion in the transplant [25, 27, 31]. However, the high costs and documentation expenditure of external tissue engineering and the requests of patients for a one‐step procedure led to growing reservations about the MACI procedure [15, 22]. To extend the minced cartilage technique for larger defect several solutions were discussed. Adding growth factors like PRP or biomaterials is a possibility [1, 26]. Furthermore, the coverage of the minced cells seemed rational to improve the environment of cell regeneration but the data is controversial [2, 16, 19, 20, 21, 23, 36]. In this context an autologous synovial tissue flap might be a promising option because it showed promising results in animal studies with high potential of differentiation [11, 17, 35]. Moreover, the harvest of a synovial flap is easy to perform, and the tissue meets all requirements for an autologous material in cell transplantation procedures [35]. However, results about usage of a synovial flap in minced cartilage techniques is missing in the literature. In present study the developed technique of the coverage of the minced transplant was described and the clinical and radiological results after 24 months were compared to the accepted injectable MACI technique in large chondral lesions. The results showed that this minced cartilage technique works effectively in these lesions and the clinical and radiological results were similar or even better compared to the MACI technique. Moreover, no modification in the rehabilitation protocol was needed. Especially the high MOCART 2.0 scores suggest that the all‐autologous concept might be crucial in achieving sufficient tissue quality in large lesions.

However, the recent literature includes studies demonstrating superior outcomes of the non‐injectable MACI technique in medium and large cartilage lesions with a follow up of 2 years and more compared to the injectable technique of present study. Saris et al., Ossendorf et al. and Brittberg et al. reported postoperative IKDC scores of 67 ± 18.7 and 65 ± 18, respectively [7, 27]. MOCART score was reported in two large prospective randomised trials with a mean of 49.8 ± 13.6 and 59.4 ± 17.3 respectively [18, 27]. In this context, the injectable MACI used in the present study must be interpreted with caution. The results presented may potentially underestimate especially the clinical effectiveness of alternative MACI techniques, which have shown similar clinical outcomes in large studies compared to the minced cartilage technique employed in this study.

Nonetheless, regarding to our study results the minced cartilage procedure can be safely expanded to large cartilage lesions when the usage of a synovial flap is performed, and it represents a promising new treatment option for large cartilage defects.

The present study certainly has several limitations. First, the small study group makes it difficult to securely evaluate clinical effectiveness of the procedure. Moreover, the MACI technique in present study was all arthroscopic injectable which is less established, especially in large defects. Other MACI techniques showed better results in recent studies Second, the follow up of 24 months restricts the ability to draw conclusions regarding the long‐term superiority of this technique over other chondrocyte transplantation procedures. Lastly, there were no histological biopsies performed to evaluate the achieved tissue quality with both techniques.

## CONCLUSION

An all‐autologous minced cartilage procedure with a synovial flap appears to be an effective treatment for large chondral lesions, with comparable outcomes to matrix‐associated chondrocyte transplantation. The superiority in some clinical outcome measure needs to be evaluated in larger cohorts.

## AUTHOR CONTRIBUTIONS

**Johanna Mayr**: Data curation; formal analysis; investigation; visualisation; writing and editing. **Franziska Warth**: Investigation; data curation; project administration. **Nicola Oehler**: Data curation; statistical analysis; writing, reviewing and editing. **Martin Majewski**: Writing; reviewing and editing. **Christoph Lutter**: Writing; reviewing and editing. **Fabian Blanke**: Conceptualisation; data curation; formal analysis; investigation; visualisation; writing and editing.

## CONFLICT OF INTEREST STATEMENT

The authors declare no conflicts of interest.

## ETHICS STATEMENT

The study was conducted in accordance with the Declaration of Helsinki and approved by the Institutional Review Board of the University of Rostock (No. A 2024‐0046). Informed consent was obtained from all individual participants included in the study.

## Data Availability

The data underlying this article will be shared on rea sonable request to the corresponding author.
